# Systematic population-based identification of *NTRK* and *RET* fusion-positive thyroid cancers

**DOI:** 10.1530/ETJ-21-0061

**Published:** 2021-12-10

**Authors:** Markus Eszlinger, Paul Stewardson, John B McIntyre, Adrian Box, Moosa Khalil, Martin Hyrcza, Konstantin Koro, Dean Ruether, Jiahui Wu, Ralf Paschke

**Affiliations:** 1 Departments of Oncology, Pathology and Laboratory Medicine, Biochemistry and Molecular Biology, and Arnie Charbonneau Cancer Institute, Cumming School of Medicine, University of Calgary, Heritage Medical Research Building, Calgary, Alberta, Canada; 2 Institute of Pathology, University Hospital Halle (Saale), Halle (Saale), Germany; 3 Department of Medical Science and Arnie Charbonneau Cancer Institute, Cumming School of Medicine, University of Calgary, Calgary, Canada; 4 Precision Oncology Hub Laboratory, Alberta Health Services, Tom Baker Cancer Centre, Calgary, Alberta, Canada; 5 Department of Pathology and Laboratory Medicine, Cumming School of Medicine, University of Calgary, Calgary, Canada; 6 Section of Medical Oncology, Department of Oncology, Cumming School of Medicine, University of Calgary, Calgary, Canada; 7 Departments of Medicine, Oncology, Pathology and Laboratory Medicine, Biochemistry and Molecular Biology, and Arnie Charbonneau Cancer Institute, Cumming School of Medicine, University of Calgary, Heritage Medical Research Building, Calgary, Alberta, Canada

**Keywords:** thyroid, cancer, radioiodine resistance, NTRK fusions, RET fusions/mutations

## Abstract

**Objective:**

The aim of the study was to identify patients with *NTRK* fusion-positive or *RET* fusion/mutation-positive thyroid cancers, who could benefit from neurotrophic tyrosine kinase receptor (NTRK) or receptor tyrosine kinase (RET) inhibitors.

**Methods:**

Patients were identified in the Calgary prospective thyroid cancer database (*N*= 482). Patients were ‘pre-screened’ with clinically available MassARRAY® BRAF test, Colon Panel, Melanoma Panel, or ThyroSPEC™. Mutation-negative tumors were ‘screened’ for *NTRK* fusions and *RET* fusions/mutations with the Oncomine™ Comprehensive Assay v3 (OCAv3).

**Results:**

A total of 86 patients were included in 1 of 2 separate analyses. Analysis A included 42 patients with radioactive iodine (RAI)-resistant distant metastases. After pre-screening, 20 *BRAF* and *RAS* mutation-negative patients underwent OCAv3 screening, resulting in the detection of 4 patients with *NTRK*fusions and 4 patients with *RET* fusions (8/20, 40% of analyzed patients). Analysis B included 44 patients, 42 with American Thyroid Association (ATA) high and intermediate risk of recurrence and 2 with medullary thyroid carcinoma. During pre-screening, 1 patient with an *NTRK* fusion, 1 patient with a *RET* fusion, and 30 patients with *BRAF* mutations were identified. The remaining 9 patients received OCAv3 screening, resulting in detection of 1 patient with an *NTRK*fusion and 1 with a* RET* fusion (4/11, 36% of analyzed patients).

**Conclusions:**

Our findings indicate a higher rate of *NTRK* fusions and *RET*fusions in patients with thyroid cancer with RAI-resistant distant metastases and ATA high or intermediate risk of recurrence. This highlights the importance of early screening to enable intervention with a NTRK or RET inhibitor.

## Introduction

The neurotrophic tyrosine kinase receptor (NTRK) inhibitors, larotrectinib and entrectinib, were approved by the United States Food & Drug Administration (FDA) for the treatment of *NTRK* fusion-positive solid tumors that are metastatic, where surgery would result in severe morbidity, or where no satisfactory alternative therapy is available. The receptor tyrosine kinase (RET) inhibitors, selpercatinib and pralsetinib, were approved by the FDA for advanced or metastatic *RET*mutation-positive medullary thyroid cancer (MTC) or *RET* gene fusion-positive thyroid cancer. The early approval of these drugs was based on phase 1/2 data (1, 2, 3, 4, 5). Patients with thyroid cancer were one of the larger patient groups in the larotrectinib basket trial, and the selpercatinib study was conducted entirely in patients with thyroid cancer (1, 3, 4, 5). Five patients with *NTRK* fusion-positive thyroid cancer were included in the entrectinib phase 1/2 trials and one had a response (6). Both larotrecinib and selpercatinib showed marked and durable overall response rates (1, 3, 4, 5). For the first time for thyroid cancer, complete response was demonstrated in 7% of the patients with *NTRK* fusion-positive, larotrectinib-treated, metastatic or locally advanced thyroid cancer, 9% of the patients with previously treated, *RET* mutation-positive, selpercatinib-treated MTC, and 5% of the patients with previously treated *RET* gene fusion-positive, selpercatinib-treated thyroid cancer (5, 7). Only 2% of selpercatinib- and larotrectinib-treated patients and 4% of entrectinib-treated patients discontinued the treatment because of treatment-related adverse events (4, 5, 6). This compares favorably with vandetanib, cabozantinib, or lenvatinib that showed drug discontinuation rates of 21, 8, and 20%, respectively (8). Further, TRK and RET inhibitors are currently being tested in clinical trials (4, 9). The very specific and selective activity profile of these drugs requires stratification of patients for these therapies based on detection of *NTRK* gene fusions and *RET* gene fusions/mutations, respectively.

The prevalence of *NTRK*fusion and *RET* fusion has been reported in several prior studies. In The Cancer Genome Atlas (TCGA) study, *NTRK* fusions and *RET* fusions were found in 1.2 and 6.8% of mostly non-metastatic thyroid cancers (10). Across 13 TCGA-analyzed tumor types, thyroid cancer displayed the highest percentage of tumors with druggable kinase fusions at 8.7% (11). In a Massachusetts General Hospital series of primary thyroid carcinoma, *NTRK* fusions were found in 3.1% of cases and 6/11 patients with *NTRK* fusions had distant metastases (12). In a series of papillary thyroid carcinomas (PTCs), *NTRK*fusions were found in 12.6% of patients and *RET* gene fusions in 14.3% (13).

The prevalence of fusions increases for metastatic/advanced patients’ WT of other aberrations. In radioactive iodine (RAI)-resistant metastatic thyroid cancers without *BRAF*, *NRAS*, *HRAS*, or *KRAS* mutations, gene fusions were previously reported in 12% of 60 samples (4 *NTRK* fusions, 2 *RET* fusions, 1 *ALK* fusion) (14). In patients with *BRAF* mutation-negative PTC and distant metastases, 67% had *ALK, NTRK*, or *RET*fusions (15). In advanced metastatic MTC, the prevalence of *RET* mutations was reported as 90% (16).

There are several recent reviews of the pros and cons for different detection methods (17, 18) and for the treatment of kinase fusion-positive cancers (19). However, appropriate and cost-effective screening algorithms to identify and stratify patients with kinase fusion-positive cancers still need to be developed for specific cancer types. A Canadian consensus for adult patients recommends *NTRK1-3* gene fusion testing at diagnosis in unresectable or metastatic/advanced patients with all thyroid histologies and at recurrence after surgery ± RAI treatment if not already performed (20). However, since resensitization for RAI treatment (21) and treatment with the RET inhibitor, selpercatinib (5), are additional options for these patients, integrated molecular stratification strategies are required that allow stratification for all currently available treatment options.

We used a stepwise, recurrence risk-adapted, prioritization approach. This approach had a stepwise application of molecular screening tests, with increasing spectrum of mutation coverage to identify patients with thyroid cancer who could benefit from these new, highly selective, and effective drugs with low adverse event rates. We report the results of this approach in a systematic, integrated, population-based study of patients with thyroid cancer. Our aim was to identify patients with *NTRK* fusion-positive thyroid cancer and *RET* fusion/mutation-positive thyroid cancer who can benefit from treatment with NTRK and RET inhibitors.

## Materials and methods

Alberta Health Services is a comprehensive, integrated, single-payer healthcare system with centralized laboratory, pathology, surgery, endocrinology, and oncology services. It has a single electronic medical record system for over 1.5 million inhabitants of the Calgary and Southern Alberta Healthcare regions. The patients for the *NTRK* fusion and *RET* fusion/mutation screening were identified in the Calgary prospective thyroid cancer database, which contains ethics approved research-consented patients with a new diagnosis of thyroid cancer in the Calgary and Southern Alberta Healthcare regions since April 2017. As of December 2020, the Calgary prospective thyroid cancer database contained 482 patients, reflecting a consenting rate of 55.9% of 862 patients with thyroid cancer since April 2017 with American Thyroid Association (ATA) high (*n*  = 87), ATA intermediate (*n*  = 126), and ATA low (*n*  = 256) recurrence risk and non-invasive follicular thyroid neoplasm with papillary-like nuclear features or MTC patients (*n*  = 13) (230 new patients/year). All new patients with thyroid cancer in the Calgary and Southern Alberta Healthcare regions are prospectively assessed for their ATA recurrence risk, TNM (tumor-nodes-metastases)/MACIS (distant metastasis, patient age, completeness of resection, local invasion, and tumor size) staging, and their indication for RAI treatment, by the thyroid cancer triage group of the University of Calgary, Division of Endocrinology. The study was approved by the University of Calgary’s ethical committee. Consent has been obtained from each patient after full explanation of the purpose and nature of all procedures used.

**Figure 1:**
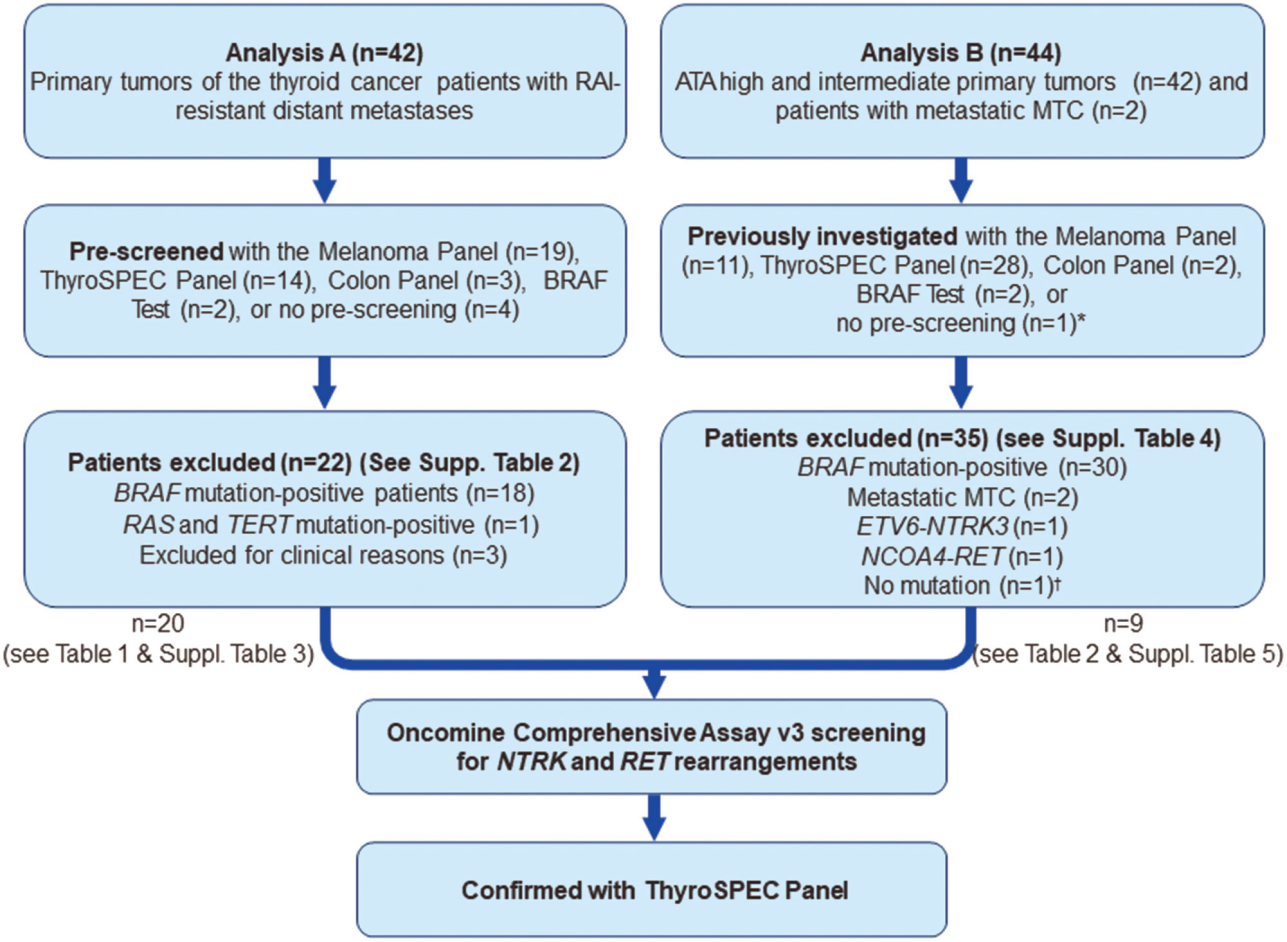
*NTRK* and *RET* gene fusion screening.

A total of 86 patients were included in 1 of 2 separate analyses we will refer to as Analysis A and Analysis B. Analysis A focused on the primary tumors of 42 patients with RAI-resistant distant metastases, identified in June 2020 (Fig. 1). These 42 patients comprise all patients with RAI-resistant distant metastases in the Calgary and Southern Alberta Healthcare regions diagnosed and either treated with Lenvatinib or observed for evidence of progression in June 2020 by the 2 medical oncologists and 2 endocrinologists treating patients with RAI-resistant distant metastases in the Calgary and Southern Alberta Healthcare regions. Sixteen patients with RAI-resistant distant metastases who were diagnosed with thyroid cancer since April 2017 were covered by the thyroid cancer database. Further 3 patients who were not consented until June 2020 and 23 patients who were diagnosed with RAI-resistant distant metastases in the Calgary and Southern Alberta Healthcare regions before April 2017 were identified by capturing all patients with thyroid cancer treated by the 2 medical oncologists and 2 endocrinologists treating patients with RAI-resistant distant metastases in the Calgary and Southern Alberta Healthcare regions.

Sixteen of the 42 patients were on treatment with lenvatinib. One patient in whom lenvatinib was discontinued was subsequently identified as *NTRK* fusion-positive. To enrich the Oncomine™ Comprehensive Assay v3 (OCAv3) screening for *NTRK* fusions and *RET* fusions/mutations, the primary tumors were first ‘pre-screened‘ with the successively clinically available MassARRAY® tests, BRAF test, Colon Panel, Melanoma Panel, or ThyroSPEC™ test, with increasing the mutation coverage respectively in addition to *BRAF* to identify *BRAF*mutation-negative patients. The mutation spectrum analyzed by these MassARRAY tests in addition to *BRAF* is given in Supplementary Table 1 (see section on supplementary materials given at the end of this article).

Analysis B targeted the primary tumors of separate 44 patients out of the 482 patients in the Calgary prospective thyroid cancer database (Fig. 1). These 44 patients included 39/87 ATA high and 3/126 ATA intermediate recurrence risk patients whose primary tumors had previously been investigated with one of the MassARRAY tests. Performance of MassARRAY for the ATA intermediate- and ATA high-risk patients was dependant on the assessment by the six members of the thyroid cancer triage group of the University of Calgary, Division of Endocrinology treating nearly all patients with thyroid cancer in the Calgary and Southern Alberta Healthcare regions. The criteria for the use of MassArray assessment evolved based on the progressive availability of the four MassArray tests described in Supplementary Table 1, case discussions during monthly interdisciplinary thyroid cancer rounds and thyroid cancer treatment pathway evolutions and updates discussed during the meetings of the interdisciplinary provincial endocrine tumor team.

In addition, 2 patients with metastatic MTC were included in these 44 patients.

### Oncomine comprehensive assay analysis

DNA and RNA were extracted from formalin-fixed paraffin-embedded (FFPE) tissues using the RecoverAll Total Nucleic Acid Isolation Kit (Thermo Fisher Scientific) according to the manufacturer’s protocol. DNA and RNA were quantified using Qubit™ DNA HS assay and Qubit RNA HS assay, respectively. Samples were sequenced using the OCAv3 Chef kit (Thermo Fisher Scientific). Briefly, amplicon libraries were prepared on the Ion Chef instrument using 20 ng DNA input and 22 ng RNA input. RNA was reverse transcribed using SuperScript VILO IV (Thermo Fisher Scientific) according to the manufacturer’s protocol. DNA and RNA fusion libraries were combined at an 80:20 ratio (DNA:RNA) and then diluted to a final concentration of 50 pM. Sample libraries were then templated and amplified on ion sphere particles and loaded on to 540 chips using the Ion 540 Chef Kit. Each 540 chip multiplexed seven samples and one no template control. Samples were sequenced on the Ion S5 XL sequencer. Data were analyzed using Ion Torrent Suite software v5.14, and variant calling was performed with Ion Reporter v5.16 Oncomine comprehensive v3 workflow 4.1. All variant calls were manually reviewed using Integrated Genomics Viewer.

### BRAF, Colon Panel, Melanoma Panel, and ThyroSPEC assay analysis

DNA and RNA were extracted from FFPE tissues using the AllPrep DNA/RNA FFPE kit (Qiagen), and RNA was reverse transcribed using Qiagen’s miScript II kit. Mutation and fusion profiling was performed with Agena Bioscience (San Diego, CA, USA) using iPLEX® Pro (ThyroSPEC, Calgary, Alberta, Canada), iPLEX® HS Colon, or iPLEX® HS Melanoma chemistry on the Agena Bioscience MassARRAY system, with all variant calls reviewed manually using Typer software. All kits were used according to the manufacturer’s protocol.

The genotyping process was performed after PCR amplification using a single-base extension technique and mass spectrometry on the MassARRAY instrument. The sensitivity of these assays is 5%, although a conservative 10% minimum frequency threshold is used for ThyroSPEC.

The genomic alterations each of these tests cover are provided at the following web addresses: Alberta Precision Laboratories | Lab Services (https://www.albertahealthservices.ca/webapps/labservices/indexAPL.asp?id=8557&tests=&zoneid=1&details=true), Agena Melanoma Panel v1.1 (https://www.albertahealthservices.ca/assets/wf/lab/if-lab-hp-cal-agena-melanoma-panel-v1-0.pdf), Agena Colon Panel v1.0 (https://www.albertahealthservices.ca/assets/wf/lab/if-lab-hp-cal-agena-colon-panel-v1.pdf), and ThyroSPEC Molecular Testing of Indeterminate FNA Cytologies (https://www.albertahealthservices.ca/assets/info/hp/cancer/if-hp-cancer-guide-molecular-fna-report.pdf).

## Results

Analysis A included 42 thyroid cancer patients with RAI-resistant distant metastases. Pre-screening of these 42 patients led to exclusion of 22 patients from further OCAv3 analysis (Fig. 1). Of these 22 patients, 18 primary tumors were *BRAF* mutation-positive, 1 had an *HRAS* mutation and a *TERT* mutation, and 3 patients were excluded because of clinical reasons (Fig. 1 and Supplementary Table 2).

**Figure 2:**
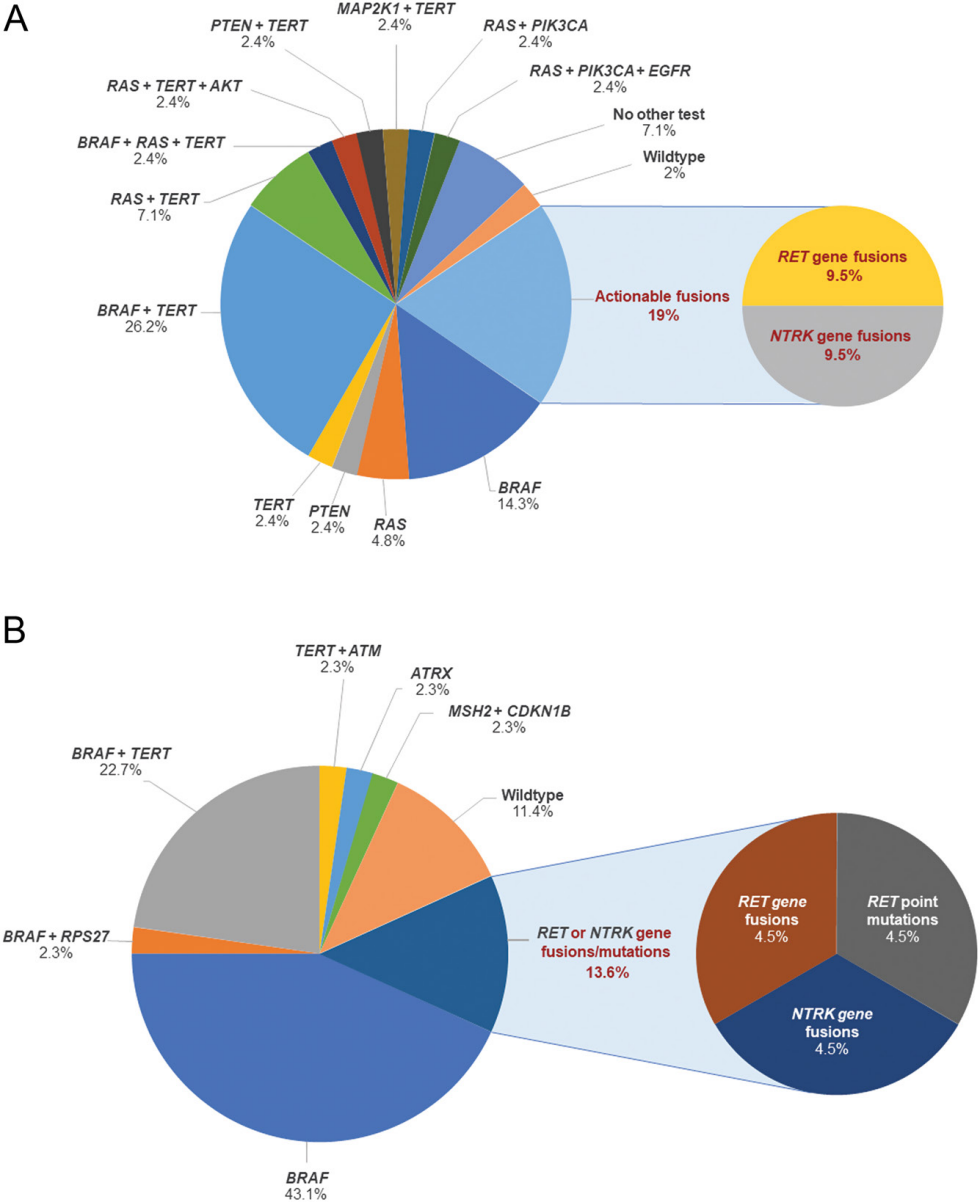
(A) Mutations and fusions detected in 42 patients with radioiodine-resistant distant metastasis. (B) Mutations and fusions detected in 42 American Thyroid Association (ATA) high and intermediate risk patients and 2 patients with medullary thyroid cancer (MTC).

This pre-screening led to the identification of 20 *BRAF* mutation-negative patients with RAI-resistant distant metastases (Fig. 1 and Supplementary Table 3) who were further analyzed using the OCAv3 panel. We detected 4 *RET* gene fusions (20%) and 4 *NTRK* fusions (20%) in the 20 *BRAF* mutation-negative patients with RAI-resistant distant metastasis in Analysis A (Table 1). Of the 42 patients with RAI-resistant distant metastases, actionable fusions were ultimately identified in 19% of patient tumors (Fig. 2).

**Table 1:** Analysis A: Result of first OCAv3^a^ analysis for 20 *BRAF* mutation-negative patients with RAI-resistant distant metastasis.

OCAv3 test results	Gene (number of mutations) (histology)
*RET* fusions (*n* = 4)	*RET* fusion only (3) *CCDC6-RET* (dsPTC^b^) *NCOA4-RET* (PTC not specified) *NCOA4-RET* (dsPTC) *NCOA4-RET*&* TERT* c.1-124C>T (1) (PTC solid variant)
*NTRK* fusions (*n* = 4)	*NTRK* fusion only (3) *SQSTM1-NTRK1*(PTC classic) *EML4-NTRK3*(mixed histology (classic and follicular variant PTC)) *ETV6-NTRK3* (PTC clear cell variant) *ETV6-NTRK3* & *MYCNOS* & *TERT* (1) (mixed histology (classic and follicular variant PTC))
Other mutations (*n* = 11)	*RAS* only (2) (FTC minimally invasive, PTC tall cell variant) *RAS* & *TERT* (2) (PTC oncocytic variant, PTC not specified) *PTEN* only (1) (PTC poorly differentiated) *TERT* only (1) (PTC oncocytic variant) *PTEN* & *TERT* (1) (Hurthle cell carcinoma) *MAP2K1* & *TERT* (1) (PTC not specified) *RAS* & *PIK3CA* (1) (FTC^c^ widely invasive) *PIK3CA* & *EGFR* & *RAS* (1) (PTC follicular variant) *RAS* & *AKT1* & *TERT* (1) (PTC poorly differentiated)
No mutation (*n* = 1)	FTC widely invasive

^a^Oncomine Comprehensive Assay v3; ^b^Diffuse sclerosing papillary thyroid cancer; ^c^Follicular thyroid cancer.

Twelve of the 87 ATA high recurrence risk patients in the database had distant metastases and were included in the Analysis A of patients with RAI-resistant distant metastases. Comparison of the 42 pre-screened patients with ATA high recurrence risk with the 36 not pre-screened ATA high recurrence risk patients (87 (ATA high-risk patients in database) − 12 (with distant metastases) − 39 (who were pre-screened) = 36 (ATA high risk recurrence, not pre-screened)) showed no significant differences for the percentage of excellent response, and the percentages of indeterminate response, structural incomplete response, and biochemical incomplete response, or the mean and median follow-up time.

Analysis B included 44 patients distinct from those included in Analysis A; 42 of these patients were ATA high and intermediate recurrence risk and 2 of these patients had metastatic MTC. Thirty-five of the 44 patients were excluded from OCAv3 analysis due to the detection of *BRAF* mutations in 30 patients and ThyroSPEC detection of 1 *ETV6-NTRK3* fusion in an ATA high-recurrence risk patient with indeterminate response to treatment at 53 months after total thyroidectomy, 1 *NCOA4-RET* gene fusion in an ATA high-recurrence risk patient with indeterminate response to treatment, and 2 *RET* point mutations (p.C620R and p.A883F), in the 2 patients with metastatic MTCs (Fig. 1 and Supplementary Table 4).

The primary tumors of the remaining nine patients in Analysis B were selected for further OCAv3 analysis (Supplementary Table 5).

**Figure 3:**
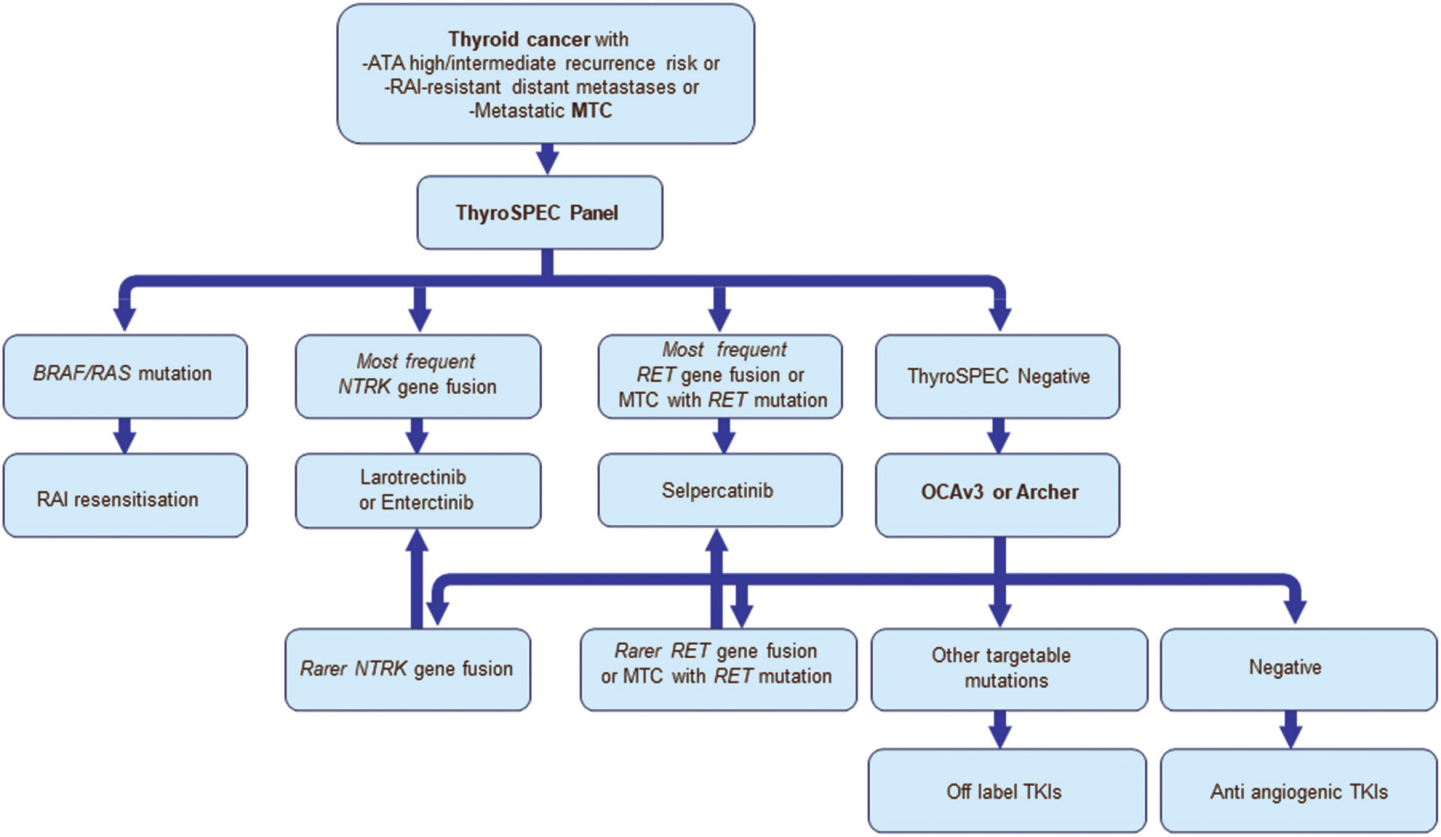
Proposed testing and treatment algorithm for Alberta Health Services.

Out of the nine patients who received OCAv3 screening in Analysis B, we detected one *NTRK*fusion (11%) and one *RET* fusion (11%) (Table 2). Of the 42 ATA high- and intermediate-risk patients and 2 patients with MTC, *RET*mutations, *RET/PTC* fusions, or *NTRK* fusions were ultimately detected in 14% (Fig. 3).

**Table 2:** Analysis B: Result of OCAv3^a^ analysis for nine patients with ATA high or intermediate recurrence risk and no *BRAF* mutation, *RET* mutation, *NTRK* fusion, or *RET* fusion in the pre-screening.

OCAv3 test results	Mutated gene (number of mutations)
*RET* fusion (*n* = 1)	*RET* fusion only (1) *CCDC6-RET*
*NTRK* fusion (*n* = 1)	*NTRK* fusion only (1) *TPM3-NTRK1* (selected based on dsPTC^b^ histology)
Other mutations (*n* = 3)	*ATRX* only (1) (PTC poorly differentiated) *MSH2* & *CDKN1B* (1) (PTC poorly differentiated) *ATM* & *TERT* (1) (PTC poorly differentiated)
No mutation (*n* = 4)	Hurthle cell carcinoma (1) PTC tall cell (1) PTC classic (1) PTC follicular variant (1)

^a^Oncomine Comprehensive Assay v3; ^b^Diffuse sclerosing papillary thyroid cancer.

All the OCAv3-detected *RET* and *NTRK* fusions detected in tumors that were pre-screened with the successively clinically available MassARRAY® tests, BRAF test, Colon Panel, and Melanoma Panel test, are covered by the ThyroSPEC assay and were subsequently confirmed with the clinically validated ThyroSPEC assay after their detection by OCAv3 analysis. One fusion-positive OCAv3-analyzed patient with RAI-resistant distant metastases was found to be ThyroSPEC mutation-negative in the pre-screening of a >15-year-old FFPE sample. Repeat ThyroSPEC testing with a more recent sample confirmed the *ETV6-NTRK3* fusion detected by OCAv3.

## Discussion

The two most important results of this systematic approach to identify patients with *NTRK* fusion and *RET* fusion-positive thyroid cancers can be summarized as follows:

1. For 20 *BRAF* mutation-negative patients, among 42 patients with RAI-resistant metastatic thyroid cancer, the OCAv3 analysis detected 4 *NTRK* fusions and 4 *RET* fusions. This 40% prevalence of *NTRK* fusion or *RET* fusion positivity for *BRAF* mutation-negative RAI-resistant metastatic thyroid cancers significantly contrasts with the previously reported 12% prevalence (4 *NTRK*fusions, 2 *RET* fusions, and 1 *ALK* fusion) for 60 *BRAF* mutation-negative patients (Pearson’s Chi-squared test, *P*  = 0.013) (14).
2. In 11 *BRAF* mutation-negative patients among 42 patients with ATA high and intermediate recurrence risk follicular thyroid cancers, the OCAv3 and ThyroSPEC analyses detected 2 *NTRK* fusions and 2 *RET* fusions (36%) (2 identified during pre-screening with ThyroSPEC and 2 identified during subsequent OCAv3 analysis).

The results of our systematic screening strategy suggest a much higher rate of *NTRK* fusions and *RET* fusions in our pre-selected patient groups than previously reported in the TCGA study with mostly non-metastatic thyroid cancers or for RAI-resistant metastatic thyroid cancers without *BRAF*, *NRAS*, *HRAS,* or *KRAS* mutations (10, 14). Our results align closer to the more recent series of patients from Lan and colleagues, where 67% of *BRAF* mutation-negative patients with PTC with distant metastases were found to have gene fusions (15).

Most important, these results suggest that all *BRAF* mutation-negative patients with RAI-resistant metastatic thyroid cancer should be screened for *NTRK* fusions and *RET* fusions to stratify these patients early for treatment with the NTRK and RET inhibitors larotrectinib, entrectinib, and selpercatinib, pralsetinib. If *NTRK* fusions and *RET* fusions are identified, these patients could benefit from higher and more sustained response rates and a much lower burden of adverse events than current standard of care.

Furthermore, this study demonstrates for the first time that all *BRAF*mutation-negative patients with progressive structural incomplete response to total thyroidectomy and RAI treatment and ATA high- or intermediate-recurrence risk patients with progressive structural incomplete response should be screened for *NTRK* fusions and *RET* fusions to enable early intervention. These patients require early stratification for cutting-edge treatment options, since 56–72 and 21–34% of patients with high or intermediate risk of recurrence patients, respectively, have persistent/recurrent structural disease a median of 4–10 years after total thyroidectomy and RAI (22, 23, 24, 25). Revision surgery and/or a second RAI treatment is largely unsuccessful and will only lead to remission in up to 51% of these patients (26, 27, 28).

Since all the OCAv3-detected *RET* fusions and *NTRK* fusions were subsequently confirmed with the ThyroSPEC Panel, future primary molecular stratifications of progressive RAI-resistant metastatic and ATA high recurrence risk thyroid cancers with structural incomplete response in the Alberta Healthcare regions will be performed with the ThyroSPEC Panel. Aberration-negative samples should still be further investigated with broader tests like OCAv3 or Archer FusionPlex Thyroid and Lung to account for the rarer fusions with *DIAPH1*, *EML4*, *IRF2BP2*, *NFASC*, and *PPL*, which were *NTRK* partners reported in the larotrectinib phase 1 and 2 trials and* ETV6, EML4, RBPMS, SQSTM1, TPM3, IRF2BP2, SQSTM1,*and *TPR* detected in a recent NTRK rearrangement detection study that are not included in the ThyroSPEC Panel and *CCDC186*, *ERC1*, *KTN1*, and *RUFY3*, which were *RET* partners reported in the selpercatinib trial not included in the ThyroSPEC Panel (4, 5, 29, 30). This approach will stratify *BRAF* mutation-positive patients for re-expression of the iodine symporter with dabrafenib and trametenib to re-enable RAI treatment. Moreover, this approach will simultaneously identify most patients eligible for treatment with larotrectinib, entrectinib and selpercatinib, pralsetinib. Thus, very few patients will require analysis with more comprehensive mutation and gene fusion panels to provide access to currently available drugs (Fig. 3).

A recent international expert consensus recommends that all patients with advanced (unresectable or metastatic) solid tumors without actionable driver gene mutations/fusions/amplifications should be tested for *NTRK* fusions, especially *ETV6-NTRK3* fusions in tumors with a high incidence of *NTRK* fusions (19). Also, patients with locally advanced tumors with a high incidence of *NTRK* fusions should be tested when considering neoadjuvant therapy before resection (19). This testing should be performed before or during the standard treatment of advanced solid tumors (19). Our results support these recommendations and add further detail to these recommendations for thyroid cancer. The Canadian consensus for adult patients recommends *NTRK1-3* fusion testing at diagnosis in unresectable or metastatic/advanced patients with all thyroid histologies and at recurrence after surgery ± RAI if not already performed (20). While these recommendations focused on NTRK inhibitors, our integrated molecular strategy allows stratification for all currently available treatment options and includes resensitization for radioiodine treatment or treatment with the RET inhibitor selpercatinib (5, 21).

Patients with metastatic dsPTC (2 of 20 OCAv3-screened patients in Analysis A and 1 of 9 OCAv3-screened patients within Analysis B) show a very high yield for *RET* fusions (2/3, 66%). This is in line with a previous report by Joung and colleagues, which demonstrated for 37 cases of dsPTC, 17 were positive for *RET/PTC1* (46%) and 6 for *RET/PTC3* (16%) (31).

The OCAv3 analysis also detected further mutations summarized in Table 3 with potential relevance for off-label drug use based on current clinical trials (clinicaltrials.gov) (32). The future treatment and follow-up of the respective patients and availability of new drugs will determine if patients with negative results for pre-screening tests like ThyroSPEC should undergo more extended mutation screening as currently done for lung cancers in the Lung Cancer Master Protocol (Lung-MAP) (33, 34) or multiple myeloma in the Bellini study (35) and other programs.

**Table 3:** Mutations detected with OCAv3^a^ with potential relevance for off-label drug use based on current clinical trials.

Mutations (VAF^b^)	ClinVar interpretation (25)	OncoKB interpretation (26)	OncoKB therapeutic effect (26)	Clinical trials
PTEN R130L (27%)	Pathogenic	Oncogenic	Compelling biological evidence supports the biomarker as being predictive of response to a drug but neither biomarker nor drug is standard of care	NCT02465060
PIK3CA H1047R (23%)	Pathogenic	Oncogenic	FDA^c^-recognized biomarker predictive of response to an FDA-approved drug in this indication (breast cancer: fulvestrant + alpelisib)	NCT02465060
EGFR P848L (52%)	Likely benign/unknown significance	Likely neutral	–	–
MSH2 Q510* (73%)	Pathogenic	Unknown oncogenic effect	No FDA-approved or NCCN^d^ compendium listed treatments specifically for patients with thyroid cancer with MSH2-truncating mutations.	–
CDKN1B S7* (8%)	Uncertain significance	Unknown oncogenic effect	No FDA-approved or NCCN-compendium listed treatments specifically for patients with CDKN1B S7 mutant thyroid cancer.	..
ATM S151* (85%)	Uncertain significance	Unknown oncogenic effect	No FDA-approved or NCCN compendium listed treatments specifically for patients with ATM S151 mutant thyroid cancer.	28 ongoing studies for patients with *ATM*-mutated solid tumors
ATRX E680* (42%)	No entry	Unknown oncogenic effect	No FDA-approved or NCCN compendium listed treatments specifically for patients with ATRX E680 mutant thyroid cancer.	NCT03718091

^a^Oncomine Comprehensive Assay v3; ^b^Variant allele frequency; ^c^Food & Drug Administration; ^d^National Comprehensive Cancer Network.

Possible limitations of this study include the lack of RNAseq as a comprehensive gold standard for rearrangement detection and the past report of concomitant *RAS* and *BRAF*or *RET/PTC* and *BRAF* mutations in 11 of 88 mostly advanced stage PTCs and in 14 of 72 conventional PTCs (36, 37). Similarly, Yoshihara and colleagues found targetable kinase mutations in 4.9% of patients with thyroid cancer and *RET* fusions, 1% of patients with thyroid cancer with *NTRK1* fusions, and 1.8% of patients with thyroid cancer with *NTRK3* fusions (11). Therefore, further data are required to decide if *BRAF-* and *RAS*-positive patients should be excluded in future screening strategies for *RET/PTC* or *NTRK* fusions. However, pre-screening with ThyroSPEC covering the most frequent *BRAF*, *RAS,* and *RET* point mutations, as well as the most frequent *RET*/PTC and *NTRK* fusions, may effectively address this question.

In summary, our results demonstrate a high frequency of *NTRK* fusions and *RET* fusions/mutations in patients with thyroid cancer with RAI-resistant distant metastases, ATA high, or ATA intermediate risk of recurrence, whose tumors are negative for other mutations/fusions. Our data emphasize the need for genomic testing in these patient populations to identify those who may benefit from effective and well-tolerated therapies, larotrectinib, entrectinib, and selpercatinib.

## Supplementary Material

Supplementary table 1. Mutation spectrum analyzed by MassARRAY tests.

## Declaration of interest

A B reports: Advisory board honoraria from AstraZeneca and Bayer. M H reports: Advisory board and teaching honoraria from Bayer. D R reports: Advisory board honoraria from Eisai, Ipsen, Novartis, Pfizer. R P reports: Grants from Bayer, and advisory board honoraria from Bayer and Eisai. The other authors have nothing to disclose.

## Funding

The work was funded by Bayer http://dx.doi.org/10.13039/100004326 Inc.

## Author contribution statement

M E: study outline and organization, data collection, verification of underlying data and discussion of data, and writing of the manuscript. P S: data collection, verification of underlying data and discussion of data, and writing of the manuscript. J B M: performed the OCAv3 analysis. A B: responsible for melanoma and Colon Panel and ThyroSPEC analysis. M K: performed histology review. M H: performed histology review. K K: performed histology review. D R: contributed patients with metastatic thyroid cancer. J W: data collection, verification of underlying data, and statistical analysis. R P: study outline and organization, data collection, verification of underlying data and discussion of data, and writing of the manuscript.
