# Supplementary material for: Systematic population-based identification of NTRK and RET fusion-positive thyroid cancers
Source: Eur Thyroid J. 2021 Dec 10;11(1):e210061. doi: 10.1530/ETJ-21-0061 (PMC9142806; doi:10.1530/ETJ-21-0061)
Supplement: Supplementary table 1. Mutation spectrum analyzed by MassARRAY tests. [file supplementary_table_1.pdf]

Supplementary table 1. Mutation spectrum analyzed by MassARRAY tests.

Panel	Mutated Gene [Number of mutations]	
**ThyroSPEC™ Panel**	*AGK-BRAF*	*PAX8-PPARG*
	AKAP-BRAF	*PIK3CA* [5]
	AKT1 [1]	*PTEN* [1]
	BRAF [7]	*RET* [19]
	CRTC1-MAML2	*RET-PTC* [4]
	CTNNB1 [11]	*RPS2P32-THADA*
	DICER1 [3]	*SND1-BRAF*
	EGFR [1]	*SPOP* [1]
	EIF1AX [4]	*SQSTM1-NTRK3*
	EML4-ALK	*STRN-ALK*
	ETV6/NTRK3	*TERT (c.1-124C>T, c.1-146C>T)*
	EZH1 [1]	*TFG-NTRK1*
	HRAS [15]	*TMEM233-PRKAB1*
	IDH1 [1]	*TP53* [10]
	IGF2BP3-THADA	*TPM3-NTRK1*
	KRAS [17]	*TPR-NTRK1*
	NRAS [15]	*TSHR [2]*
**Melanoma Panel**	*BRAF* [22]	*NRAS* [19]
	GNA11 [3]	*PTEN* [5]
	GNAQ [3]	*RAC1* [1]
	HRAS [3]	*RPS27* [1]
	KIT [51]	*TERT* (c.1-124C>T, c.1-146C>T)
	KRAS [5]	
**Colon Panel**	*BRAF* [3]	*NRAS* [30]
	EGFR [2]	*PIK3CA* [4]
	KRAS [46]	
**BRAF Test**	(V600E/V600E complex (V600Ec))	(V600K)
	(V600D)	(V600R)

Supplementary table 2. Analysis A: Mutation pre-screening results for 22 primary tumors of patients with RAI[1]-resistant distant metastasis excluded from OCAv3[2] analysis.

**Panel**	**Mutated Gene [Number of Mutations]**
**Melanoma Panel (n=12)**	*BRAF* & *TERT* [9]<br>*BRAF* only [1]<br>*BRAF* & *TERT* & *RAS* [1]<br>*HRAS* & *TERT* [1]
**ThyroSPEC™ Panel (n=4)**	*BRAF* only [2]<br>*BRAF* & *TERT* [2]
**Colon Panel (n=3)**	*BRAF* only [3]
**No Other Test (n=3)**	Selected based on dsPTC[3] histology

[1] radioactive iodine
[2] Oncomine Comprehensive Assay v3
[3] diffuse sclerosing papillary thyroid cancer

Supplementary table 3. Analysis A: Mutation pre-screening results for 20 primary tumors of patients with RAI[1]-resistant distant metastasis selected for OCAv3[2] analysis.

Panel	Mutated Gene [Number of Mutations]
**ThyroSPEC™ Panel (n=10)**	*TPR-NTRK1* fusion [1]
	RAS only [1]
	RAS & *IDH1* [1]
	RAS & *PIK3CA* [1]
	NCOA4-RET fusion [1]
	TERT only [2]
	no mutation [3]
**Melanoma Panel (n=7)**	*RAS* & *TERT* [4]
	TERT only [2]
	no mutation [1]
**BRAF Test (n=2)**	no mutation [2]
**No Other Test (n=1)**	selected based on dsPTC[3] histology

[1] radioactive iodine
[2] Oncomine Comprehensive Assay v3
[3] diffuse sclerosing papillary thyroid cancer

Supplementary table 4. Analysis B: Mutation pre-screening results for 33 ATA[1] high and intermediate recurrence risk patients and two patients with metastatic MTCs[2] excluded from OCAv3[3] analysis.

Panel	Mutated Gene [Number of Mutations] (Histology)
**ThyroSPEC™ Panel (n=22)**	*BRAF* only [13]
	BRAF & *TERT* [4]
	RET only [2]
	NCOA4-RET
	RET (MTC)
	RET & *IDH1* [1] (MTC)
	NTRK only [1]
	ETV6-NTRK3
	No mutation [1]*
**Melanoma Panel (n=10)**	*BRAF* only [3]
	BRAF & *RPS27* [1]
	BRAF & *TERT* [6]
**BRAF Test (n=2)**	*BRAF* only [2]
**Colon Panel (n=1)**	*BRAF* only [1]

*Patient was previously identified as OCAv3 *BRAF* mutation-positive and was excluded from further OCAv3 analysis.

[1] American Thyroid Association
[2] medullary thyroid cancer
[3] Oncomine Comprehensive Assay v3

Supplementary table 5. Analysis B: Mutation pre-screening results for 9 ATA[1] high and intermediate recurrence risk patients selected for OCAv3[2] analysis.

**Panel**	**Mutated Gene [Number of Mutations]**
**ThyroSPEC™ Panel (n=6)**	*TERT* only [1]<br>No mutation [5]
**Melanoma Panel (n=1)**	No mutation [1]
**Colon Panel (n=1)**	No mutation [1]
**No Other Test (n=1)**	Selected based on dsPTC[3] histology

[1] American Thyroid Association
[2] Oncomine Comprehensive Assay v3
[3] diffuse sclerosing papillary thyroid cancer
